# Editorial: Quality and quantity in research assessment: Examining the merits of metrics

**DOI:** 10.3389/frma.2022.991550

**Published:** 2022-08-12

**Authors:** Maziar Montazerian, Bertil Fabricius Dorch

**Affiliations:** ^1^Academic Unit of Materials Engineering, Federal University of Campina Grande, Campina Grande, Brazil; ^2^University Library of Southern Denmark, University of Southern Denmark (SDU), Odense, Denmark; ^3^Department of Physics, Chemistry and Pharmacy, University of Southern Denmark (SDU), Odense, Denmark

**Keywords:** metrics, bibliometrics, research assessment, research ethics, research policy

The staggering growth of published data emphasizes the importance of reliable bibliometric/scientometric metrics when analyzing a research field's or researchers' performance as qualified by their research output. Bibliometric/scientometric analyses are widely employed nowadays and have become common tools e.g., for systematic literature studies that evaluate scientific progress, future trends or the identification of research gaps. Such analyses may use statistical methods to analyze publications, books, articles and other resources regarding scientific content, quantity and sometimes quality (Waltman, 2016; Montazerian et al., 2017). For example, in this Research Topic, bibliometric/scientometric studies have revealed a global upsurge in nanotechnology [Idamokoro and Hosu (a)], post-COVID multidisciplinary research (Mondal et al.), and waste materials in livestock food production [Idamokoro and Hosu (b)], which are important for the world's sustainable growth. After thoroughly investigating the data on literature and harnessing statistical tools, valuable advice for collaboration between countries, emerging themes, recent research directions, and challenges ahead of these specific topics are provided. The bibliometrics/scientometrics can offer several good insights on where and how the science is researched. However, they are just a tool that can complement expert panels. The problem with the use of one single indicator by funding agencies and institutions has led to an epidemic of academic publishing manifested by associating the academic performance of individuals with the number of publications/citations (quantity) than with the content of their works (quality) (Montazerian et al., 2019).

The publication mania is causing significant ambiguity in the way science is done and in how scientists' performance is evaluated. For example, single scientometric indicators, such as the h-index or journal impact factor (JIF) in scientific assessments, are widely used by agencies. They can cause multiple issues because such indices typically do not usually consider the age or career stage, the field size, publication and citation cultures in different areas, co-authorship, etc. Although the number of publications/citations, h-index, JIF, and so forth are relevant and may be taken as a measure of visibility and popularity, they are certainly not indications of neither intellectual value nor of scientific quality (Montazerian et al., 2020).

To judge the quantity vs. quality, some researchers dwell on rigorous and complementary indicators of a scientists' performance by critically analyzing a plethora of scientometric data. Some have argued that the scientific performance of an individual or group must be evaluated by peer review processes based on their impact in their fields or the originality, strength, reproducibility, and relevance of their publications. For example, Põder has tried to address many problems of contemporary evaluative bibliometrics that are unable to include the effect of all factors, e.g., multiple authorship. He recommended establishing theoretically sound and practical holistic indicators by combining several sub-indices. This requires developing fractionalized indicators readily available in well-known databases and informing the scientific community more clearly about their meaning and purpose. The application of this all-inclusive index is indispensable. It demands further effort by the community to popularize such indices because traditional indicators such as the h-index have become part of our research culture and many researchers and organizations find any changes detrimental to their interests. Pourret et al. have also recommended that the inclusion of diversity and equity along with a movement toward an actual open science could also help with focusing on the research and quality rather than where research is published and how many times it is cited.

These are only a few examples of endeavors toward developing holistic evaluations. Unfortunately, in an imperfect world, scientific project reviews, grant funding decisions, and university career advancement steps are often based on decisive input from non-experts who can readily use bibliometric indices. Therefore, the newer and more robust tools or methods that consider the normalization of bibliometric indices and fragmentation by the field and other influential parameters are encouraged to be shared and embraced by the research community, universities, and funding agencies. In addition, we need to investigate whether high quantity also implies high quality/significance/reputation. The role of peer review or in-depth studies in highlighting the quality based on the originality, strength, reproducibility, and relevance of the publications could also be an exciting topic for future studies.

We finish the editorial by referring to the recent European Commission's scoping report confirming that “publish or perish” culture is damaging for research and researchers as the current state of research evaluation in Europe primarily relies on the races for publications and citations at the expense of quality. The commission encourages the funding agencies to sign an agreement to effectively ensure that their research assessments will recognize and reward the plurality of contributions of researchers to academic life (not just publishing and bringing in grant money). Research assessment organizations should respect epistemic differences between research fields. They should reward new (or newly emphasized) quality dimensions such as open science (broadly defined), research integrity, and societal relevance when evaluating individuals, institutions and research proposals^1^,^2^.

## Author contributions

Both authors listed have made a substantial, direct, and intellectual contribution to the work and approved it for publication.

## Conflict of interest

The authors declare that the research was conducted in the absence of any commercial or financial relationships that could be construed as a potential conflict of interest.

## Publisher's note

All claims expressed in this article are solely those of the authors and do not necessarily represent those of their affiliated organizations, or those of the publisher, the editors and the reviewers. Any product that may be evaluated in this article, or claim that may be made by its manufacturer, is not guaranteed or endorsed by the publisher.

## Footnotes

^1^ https://www.leidenmadtrics.nl/articles/what-lies-ahead-for-research-assessment-reforms-in-europe

^2^ https://www.consilium.europa.eu/en/press/press-releases/2022/06/10/council-provides-political-orientations-on-international-cooperation-open-science-and-european-missions/
